# Age-related changes in body composition in a sample of Czech women aged 18–89 years: a cross-sectional study

**DOI:** 10.1007/s00394-013-0514-x

**Published:** 2013-04-11

**Authors:** Aleš Gába, Miroslava Přidalová

**Affiliations:** Department of Natural Sciences in Kinanthropology, Faculty of Physical Culture, Palacký University Olomouc, Tř. Míru 115, 771 11 Olomouc, Czech Republic

**Keywords:** Body fat mass, Fat-free mass, Visceral adipose tissue, Percentiles, InBody 720

## Abstract

**Background:**

The Czech Republic lacks body composition data for women. Therefore, the purpose of the study was to analyze body composition [body fat mass (BFM), fat-free mass (FFM), body fat percentage (%BFM) and visceral adipose tissue (VAT)] and to evaluate the changes that occur with aging in women aged 18–89 years. We also analyzed anthropometric characteristics of study participants and developed age-specific percentile curves for body composition parameters.

**Methods:**

A cross-sectional, non‐randomized study was conducted with a sample of 1,970 apparently healthy Czech women. Body composition was measured using a direct segmental multi-frequency bioelectrical impedance analysis (BSM-BIA).

**Results:**

The mean BFM was 19.7 ± 8.9 kg, and BFM reached its peak in women over 70, at 27.6 ± 8.8 kg. There was a strong correlation between BFM and age (*r* = 0.61; *r* ^2^ = 0.37). Fifty percent of the women in the study had a BFM between 13.0 and 25.0 kg. The %BFM (*r* = 0.69; *r* ^2^ = 0.47) and VAT (*r* = 0.88; *r* ^2^ = 0.77) were also significantly associated with age. The reference range for %BFM was 22.0–35.6 % (25th–75th percentile). The mean FFM was 45.8 ± 5.5 kg, and FFM decreased with age (*r* = −0.27; *r* ^2^ = 0.07).

**Conclusions:**

The results presented in this study showed a statistically significant increase in BFM,  %BFM and VFA as age increased, and the values reached their peak in women over 70. Even when FFM decreased slightly with age, body weight increased because of the increase in BFM.

## Introduction

Aging involves a group of processes that occur in living organisms, and it is associated with morphological, structural and functional alterations. These changes occur at the cellular level, the tissue level and the whole-body level. They affect internal stability and increase the risk of many common chronic and aging-associated diseases, including diabetes mellitus type 2, cardiovascular disease, respiratory diseases, several types of cancer, osteoporosis and Alzheimer’s disease. Age-related changes also include changes in body composition. A decrease in fat-free mass (FFM) and an increase in body fat mass (BFM) are both considered hallmarks of human aging [1] and can be used to assess functional status, disability and mortality [2]. Among women, age-related changes in body composition have been observed particularly after menopause [3–5].

FFM usually increases as humans grow, remains relatively stable throughout maturity and declines during senescence. Generally, FFM peaks between the fourth and fifth decades of life [6–8], occasionally earlier [9], and then declines slightly. The decrease in FFM primarily occurs as a result of losses in muscle mass, component of FFM, and is considered the most constant marker of aging. Moreover, the decline in muscle strength caused by the loss of muscle mass contributes to the decline in physical function, as well as increasing disability, frailty and loss of independence [10, 11].

In contrast to FFM, the amount of BFM usually increases throughout the lifespan. It generally peaks between the fifth and seventh decades of life and then remains constant or decreases slightly [3, 6–9]. Although the amount of BFM is caused by genetic factors [12], it is influenced by non-genetic factors, such as physical inactivity [13] combined with an increased intake of energy-dense foods that are high in lipids or carbohydrates. With advancing age, excess BFM is associated with obesity, and it is a strong but modifiable risk factor for all-cause mortality in both males and females [14]. According to the latest World Health Organization estimates, obesity has more than doubled since 1980. Worldwide, more than 1.4 billion adults (aged 20 years and over) are overweight. Of these individuals, over 200 million males and nearly 300 million females are obese [15].

Over the past two decades, the socioeconomic transition in the Czech Republic has caused sweeping changes in lifestyle, including more energy intake and a more sedentary lifestyle, which have contributed to an increase in the prevalence of overweight and obesity. Unfortunately, few studies on the body composition of the Czech population have been published since the 1990s, and none of them have reported possible reference values and age-related trends in Czech women aged 18 years and older. However, these data are necessary not only to evaluate the current situation but also to establish national guidelines and evidence-based prevention programs because the Czech Republic is among the European countries with the highest prevalence of overweight and obesity [16]. Therefore, the purpose of the study was to analyze body composition (BFM, FFM, %BFM and VAT) using BSM-BIA and to evaluate the changes that occur with aging in a sample of 1,970 Czech women aged 18–89 years. We also analyzed anthropometric characteristics of study participants and developed age-specific percentile curves for body composition parameters.

## Methods

### Subjects and study design

The total sample for this cross-sectional study included 2,333 apparently healthy Czech women aged 18–89 years. All of the subjects were volunteers. We eventually excluded the data from 363 of the participants due to technical causes or recording failures. Participants were also excluded from the study sample if they were 90 years of age or older or had a very low or very high physical activity level (e.g., subjects with motor skills disorders and paralysis or highly trained athletes). Hence, the present analysis is based on the data from 1,970 women. All the subjects were non-randomly recruited by responding to an offer of free body composition analysis for students (pregradual and postgradual students, and students of the University of Third Age) and university staff across the Czech Republic. The offer was publicized at exhibitions and university open-house days, as well as through invitations sent to leisure clubs for the elderly.

The purpose and risks of the study were explained to each participant before the examination, and informed written consent was obtained from all participants. The study design was approved by the Faculty of Physical Culture Ethics Committee at the Palacký University Olomouc. The research was accomplished between 2008 and 2011.

### BSM-BIA

Selected body composition parameters were examined using the InBody 720 (Biospace Co., Ltd.; Seoul, Korea) body composition analyzer, which is a tool for assessing whole and segmental body composition that has been validated against dual energy X‐ray absorptiometry [17]. The BSM-BIA device measures resistance in broadband frequencies (1, 5, 50, 250, 500 and 1,000 kHz) and reactance in mean frequencies (5, 50 and 250 kHz). All the measurements were carried out with an alternating current of 90 μA (1 kHz) and 400 μA (other frequencies). Total body impedance values were calculated by summing the segmental impedance values that were analyzed separately with a tetrapolar eight-point tactile electrode system. The surface of the hand electrode was placed in contact with each of the five fingers (the thumb was placed lightly on the thumb electrode, and the other four fingers were placed along the bottom of the palm electrode), while the participant’s heels were placed on the circular rear sole electrode before the forefoot hit the front sole electrode. In accordance with the manufacturer’s guidelines, the participants held out their arms and legs so that they would not come into contact with any other body segments during the procedure. The arms were held at approximately 20° away from the trunk, and legs were positioned 45° apart. The procedure took approximately 2 min (research mode was activated) and it required no specific skills. Before the measurements were taken, the participant’s identification number, name and surname, body height, age and sex were entered into the manufacturer’s software, Lookin’ Body, version 3.0 (Biospace Co., Ltd.; Seoul, Korea). If possible, the subjects were asked to fast for 2 h, to avoid any vigorous physical activity for at least 48 h before the procedure and to urinate or defecate before the measurements were taken.

FFM was estimated by dividing total body water (TBW) by a constant (FFM = TBW × 0.732). TBW is the sum of extracellular fluid (estimated by low-level frequencies, i.e., 1–50 kHz) and intracellular fluid (estimated by high-level frequencies, i.e., ≥250 kHz). Bedogni et al. [18] concluded that the tetrapolar eight-point tactile electrode impedance method is precise and provides accurate estimates of TBW in healthy subjects. BFM was calculated as the difference between body weight and FFM. For subsequent analyses of body composition, an average %BFM was used. VAT was represented by the visceral fat area (VFA; cm^2^), which was defined as a cross-sectional area of visceral fat in the abdomen at the umbilical level. Ogawa et al. [19] noted that BSM-BIA measured by the InBody 720 shows to be useful as a more convenient substitute for computed tomography when measuring VFA.

### Anthropometric measurements

A standard procedure was used to measure body height to the nearest 0.5 cm using a portable anthropometer P-375 (Trystom; Olomouc, Czech Republic). Body height was measured before the body composition analysis, while the subjects were wearing light clothes and no shoes. Body weight was measured with the DSM-BIA device, recorded as the total body mass rounded to the nearest 0.1 kg. BMI was calculated by dividing body weight (kg) by body height squared (m^2^).

### Statistical methods

All analyses were carried out using the statistical program Statistica, version 10.0 (StatSoft, Inc.; Tulsa, USA). The descriptive statistics were expressed as the mean ± standard deviation (SD), with a 95 % confidence interval (CI). For the statistical analyses, the study sample of 1,970 women aged 18–89 years was divided into the following age groups: 18–29 years (*n* = 962), 30–39 years (*n* = 113), 40–49 years (*n* = 108), 50–59 years (*n* = 197), 60–69 years (*n* = 437) and older than 70 years (*n* = 153).

Seven age-specific percentiles (i.e., 5th, 10th, 25th, 50th, 75th, 90th and 95th) were calculated for each body composition variable. The *i*th percentile (P*i*) was the value that *i* % of the sample fell at or below. The 50th percentile also represents the median and difference between 75th and 25th percentile correspond with the interquartile range. The construction of the percentile curves was performed using age-specific percentiles, and the raw percentile curves (5th–95th) were smoothed using a least squares method.

Kolmogorov–Smirnov test was used for checking for the normality of data distribution. We used one-way analysis of variance (ANOVA) with post hoc Tukey’s honestly significant difference (HSD) test for normally distributed variables and Kruskal–Wallis ANOVA with multiple comparisons for non-normally distributed variables to determine the significance of the difference among the age groups and between each age group with the preceding group. Eta-squared (*η* ^*2*^) was calculated via an ANOVA [*η* ^*2*^ = SS_factor_/(SS_factor_ + SS_error_)] or via Kruskal–Wallis ANOVA [*η* ^*2*^ = *H*/(*n* − 1)]. The values of 0.01, 0.06 and 0.14 were interpreted as small, medium and large effect sizes [20], respectively. A polynomial regression analysis was used to determine strength of the relationship between age (independent variable) and selected body composition variables (dependent variables). Statistical significance was set at the 0.01 probability level (*p* < 0.01), unless otherwise stated.

## Results

### Body height, body weight and BMI

The mean age of the study sample was 40.4 years. Additional descriptive statistics about the study sample and each age group are presented in Table 1. The mean body height of the sample was 164.7 ± 7.2 cm (95 % CI 164.4–165.0 cm), and the mean body weight was 65.5 ± 11.1 kg (95 % CI 65.0–66.0 kg). The statistical analysis confirmed that the differences in body height between the observed age groups were statistically significant (*F* = 118.7, *p* < 0.01; *η* ^*2*^ = 0.23). The youngest subjects were the tallest, and body height decreased among the older age groups. Additional analysis showed that women aged 50–59 years [difference (diff) = 4.3 cm, *p* < 0.01] and older than 70 years (diff = 4.1 cm, *p* < 0.01) were significantly shorter than the preceding age group.

**Table 1:** Anthropometric characteristics of study participants

	*n*	Mean ± SD	95 % CI
Body height (cm)			
All age groups	1,970	164.7 ± 7.2*^,§^	164.4–165.0
18–29 years	962	167.5 ± 6.4	167.1–167.9
30–39 years	113	166.4 ± 6.4	165.2–167.6
40–49 years	108	166.5 ± 5.9	165.4–167.6
50–59 years	197	162.2 ± 6.4^†^	161.3–163.1
60–69 years	437	161.2 ± 6.0	160.7–161.8
>70 years	153	157.1 ± 6.6^†^	156.1–158.2
Body weight (kg)			
All age groups	1,970	65.5 ± 11.1*^,§^	65.0–66.0
18–29 years	962	61.3 ± 8.4	60.7–61.8
30–39 years	113	63.8 ± 10.3	61.9–65.7
40–49 years	108	69.3 ± 12.7^‡^	66.9–71.7
50–59 years	197	70.6 ± 13.5	68.8–72.6
60–69 years	437	70.5 ± 10.7	69.5–71.5
>70 years	153	69.6 ± 11.5	67.8–71.4
BMI (kg/m^2^)			
All age groups	1,970	24.2 ± 4.3*^,§^	24.0–24.4
18–29 years	962	21.8 ± 2.5	21.6–22.0
30–39 years	113	23.1 ± 3.7^‡^	22.4–23.7
40–49 years	108	25.0 ± 4.2^†^	24.2–25.8
50–59 years	197	26.8 ± 4.8^‡^	26.2–27.5
60–69 years	437	27.1 ± 3.9	26.8–27.5
>70 years	153	28.2 ± 4.3	27.5–28.9

*n* number of participants, *SD* standard deviation, *CI* confidence interval, *BMI* body mass index

* *p* < 0.01, one-way ANOVA or Kruskal–Wallis ANOVA

^‡^ *p* < 0.05 and ^†^ *p* < 0.01, versus preceding age group, post hoc Tukey’s HSD test after one-way ANOVA or multiple comparisons by Kruskal–Wallis ANOVA

^§^ *η* ^*2*^ > 0.14

The differences in body weight between the observed age groups were also significant (*H* = 312.2, *p* < 0.01; *η* ^*2*^ = 0.16); body weight was generally greater in older participants compared to younger participants. A significant increase in body weight was detected between women 30–39 years old and women 40–49 years old (diff = 5.5 kg, *p* < 0.05). The mean BMI increased from 21.8 ± 2.5 kg/m^2^ (95 % CI 21.6–22.0 kg/m^2^) in the youngest age group to 28.2 ± 4.3 kg/m^2^ (95 % CI 27.5–28.9 kg/m^2^) in subjects over 70. BMI was only in the normal range for women 18–29 and 30–39 years old, whereas it was in the overweight range for older subjects.

### BFM and FFM

**Fig. 1:**
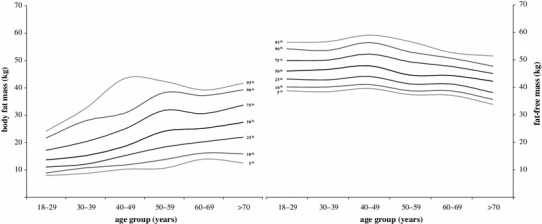
Percentile curves for BFM and FFM. Each of the raw percentile curves (5th–95th) was smoothed by least of squares method

The BFM and FFM values, categorized by age and distributed into percentiles from the 5th percentile to the 95th percentile, are given in Table 2. Additionally, Fig. 1 presents each of the raw percentiles as an age-specific smoothed curve. The mean BFM was 19.7 ± 8.9 kg (95 % CI 19.3–20.1 kg) for all age groups and significantly increased with age (*r* = 0.61; *r* ^2^ = 0.37) (Fig. 4) at an average rate of 2.58 kg per decade. The results of the Kruskal–Wallis ANOVA showed significant differences in BFM between all the age groups (*H* = 787.3, *p* < 0.01; *η* ^*2*^ = 0.40), with particularly notable differences between the participants 18–29 and 30–39 years old (diff = 2.6 kg, *p* < 0.05), 30–39 and 40–49 years old (diff = 3.7 kg, *p* < 0.05) and 40–49 and 50–59 years old (diff = 4.3 kg, *p* < 0.01). Fifty percent of the participants had BFM values less than 13.0 kg (<50th percentile) or higher than 25.0 kg (>75th percentile).

**Table 2:** Percentile values for BFM and FFM in various age categories

	*n*	Mean ± SD	95 % CI	Δ^a^ (%)	Percentile						
					5th	10th	25th	50th	75th	90th	95th
Body fat mass (kg)											
All age groups	1,970	19.7 ± 8.9*^,§^	19.3–20.1		8.7	10.3	13.0	17.6	25.0	32.5	37.4
18–29 years	962	14.7 ± 5.3	14.3–15.0		8.0	8.9	11.1	13.7	17.2	21.7	24.2
30–39 years	113	17.3 ± 7.2^‡^	15.9–18.6	18	8.7	10.8	12.1	15.2	20.3	28.0	32.5
40–49 years	108	21.0 ± 8.9^‡^	19.3–22.7	22	10.3	11.8	15.3	18.7	25.0	30.8	43.5
50–59 years	197	25.3 ± 9.6^†^	24.0–26.7	21	10.6	13.8	18.4	24.2	31.8	38.3	42.4
60–69 years	437	25.9 ± 7.9	25.2–26.6	2	14.0	16.2	20.3	25.2	30.6	37.2	39.2
>70 years	153	27.6 ± 8.8	26.2–29.0	6	12.6	15.9	22.0	27.4	33.7	39.3	41.7
Fat-free mass (kg)											
All age groups	1,970	45.8 ± 5.5*	45.5–46.0		37.5	39.1	42.0	45.4	49.0	53.0	55.7
18–29 years	962	46.6 ± 5.3	46.3–47.0		38.7	40.1	43.0	45.9	49.7	54.0	56.4
30–39 years	113	46.5 ± 5.7	45.5–47.6	0	38.4	40.1	42.7	46.5	49.9	53.4	56.6
40–49 years	108	48.4 ± 5.9	47.2–49.5	4	39.6	41.0	44.0	47.9	52.1	56.3	59.0
50–59 years	197	45.4 ± 5.8^†^	44.5–46.2	−6	37.3	38.7	41.2	44.4	49.2	52.8	56.6
60–69 years	437	44.6 ± 4.7	44.2–45.1	−2	37.1	38.7	41.2	44.3	47.6	50.6	52.6
>70 years	153	42.0 ± 5.2^†^	41.2–42.9	−6	33.8	35.6	38.1	42.2	45.0	47.7	51.4

*n* number of participants, *SD* standard deviation, *CI* confidence interval

* *p* < 0.01, one-way ANOVA or Kruskal–Wallis ANOVA

^‡^ *p* < 0.05 and ^†^ *p* < 0.01, versus preceding age group, post hoc Tukey’s HSD test after one-way ANOVA or multiple comparisons by Kruskal–Wallis ANOVA

^§^ *η* ^*2*^ > 0.14

^a^Percent difference versus preceding age group

The mean FFM was 45.8 ± 5.5 kg (95 % CI 45.5–46.0 kg) and decreased with age (*r* = −0.27; *r* ^2^ = 0.07) (Fig. 4) at an average rate of 0.92 kg per decade. This age-related trend was also apparent in the smoothed percentile curves that are shown in Fig. 1. The observed women aged 18–89 years showed a relatively flat 50th percentile varying between 42.2 and 47.9 kg over the entire age range with the peak at fourth decade. The 25th–75th percentile range for FFM was 42.0–49.0 kg. Among the younger subjects, the mean FFM changed marginally, but it declined significantly among the women 50–59 years old (diff = 3.0 kg, *p* < 0.01) and the women over 70 (diff = 2.6 kg, *p* < 0.01) in comparison with the preceding age groups.

**Fig. 2:**
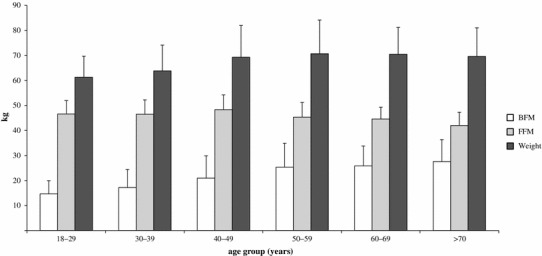
Body weight, BFM and FFM by age group. *Bars* are presented as mean ± SD

Although FFM decreased with age, body weight increased slightly because of the increase in BFM (Fig. 2). This trend was particularly apparent in the subjects aged 50–59 years; the body weight of this group was 1.3 kg greater than the weight of the women aged 40–49 years. Although FFM declined by 3.0 kg compared to the preceding age group, the increase in body weight was caused by a 4.3 kg increase in BFM. However, body weight slightly decreased among women over 60 because the decline in their FFM was greater than the increase in their BFM.

### %BFM and VAT

**Fig. 3:**
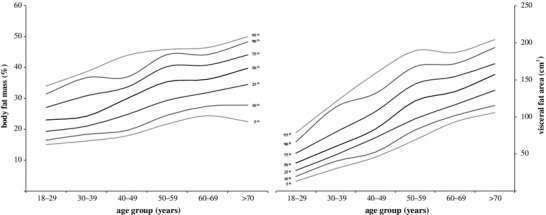
Percentile curves for the %BFM and visceral fat area. Each of the raw percentile curves (5th–95th) was smoothed by least of squares method

**Fig. 4:**
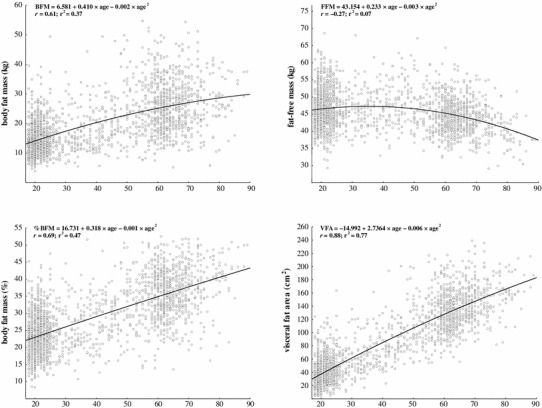
Body composition variables regressed (polynomial regression) versus age in women aged 18–89 years

As shown in Table 3, there was a statistically significant change in the %BFM over the entire age range (*H* = 921.7, *p* < 0.01; *η* ^*2*^ = 0.47). We also found a statistically significant relationship between %BFM and age (*r* = 0.69; *r* ^2^ = 0.47) (Fig. 4). The mean %BFM was 29.1 ± 8.9 % (95 % CI 28.7–29.5 %) for all age groups and ranged from 23.5 % in the youngest age group to 38.8 % in the oldest subjects. Furthermore, fifty percent of study sample had a %BFM between 22.0 and 35.6 % (25th–75th percentile). Five percent (>95th percentile) of all participants had a %BFM greater than 44.6 % (range 34.0–50.0 %), and an additional five percent (<5th percentile) had a %BFM under 16.2 % (range 15.0–24.4 %) (Table 3, Fig. 3). According to multiple comparisons by Kruskal–Wallis ANOVA, the %BFM significantly increased in comparison with the preceding age group in 30–39 years old by 2.8 % and in 50–59 years old by 5.4 %.

**Table 3:** Percentile values for the %BFM and visceral fat area in various age categories

	*n*	Mean ± SD	95 % CI	Δ^a^ (%)	Percentile						
					5th	10th	25th	50th	75th	90th	95th
Body fat mass (%)											
All age groups	1,970	29.1 ± 8.9*^,§^	28.7–29.5		16.2	18.5	22.0	28.2	35.6	41.6	44.6
18–29 years	962	23.5 ± 5.9	23.1–23.9		15.0	16.5	19.3	23.0	27.0	31.4	34.0
30–39 years	113	26.3 ± 7.2^‡^	24.9–27.6	12	16.2	18.4	21.0	24.2	30.8	36.7	38.6
40–49 years	108	29.3 ± 7.4	27.9–30.7	11	17.9	19.6	24.7	29.9	33.6	36.9	43.9
50–59 years	197	34.7 ± 7.7^†^	33.7–35.8	19	21.7	24.5	29.3	35.4	40.3	44.2	45.8
60–69 years	437	36.0 ± 6.5	35.4–36.6	4	24.4	27.5	31.8	36.2	40.8	44.2	46.4
>70 years	153	38.8 ± 7.7	37.6–40.0	8	22.5	27.8	34.5	39.7	44.1	48.3	50.0
Visceral fat area (cm^2^)											
All age groups	1,970	83.4 ± 52.2*^,§^	81.1–85.7		19.7	25.6	37.9	68.5	125.6	158.1	173.1
18–29 years	962	41.1 ± 20.1	39.8–42.4		13.3	19.7	27.8	37.8	50.9	66.2	78.5
30–39 years	113	67.0 ± 27.4^†^	61.9–72.1	63	30.2	40.2	48.7	60.1	79.5	113.3	120.3
40–49 years	108	91.6 ± 33.5^‡^	85.2–98.0	37	45.7	52.5	71.9	83.6	107.2	131.6	158.7
50–59 years	197	122.5 ± 34.2^†^	117.6–127.3	34	69.0	82.5	97.5	121.7	144.6	167.9	189.5
60–69 years	437	135.8 ± 28.3	133.2–138.5	11	93.3	101.9	116.4	134.2	154.4	171.7	186.8
>70 years	153	155.3 ± 30.3	150.4–160.1	14	105.9	115.4	136.0	157.2	171.8	193.6	204.3

*n* number of participants, *SD* standard deviation, *CI* confidence interval

* *p* < 0.01, one-way ANOVA or Kruskal–Wallis ANOVA

^‡^ *p* < 0.05 and ^†^ *p* < 0.01, versus preceding age group, post hoc Tukey’s HSD test after one-way ANOVA or multiple comparisons by Kruskal–Wallis ANOVA

^§^ *η* ^*2*^ > 0.14

^a^Percent difference versus preceding age group

Figure 4 shows a statistically significant relationship between VFA and age for women aged 18–89 years. VFA was greater in older participants than in younger participants; it increased with age (*r* = 0.88; *r* ^2^ = 0.77), according to the polynomial regression analysis. The percentile curves (Fig. 3) also demonstrated this age-related trend. Each year of increasing age was associated with a mean increase in VFA of 2.28 cm^2^. In the overall statistical analysis, the Kruskal–Wallis ANOVA showed that there was a statistically significant difference between all the age groups (*H* = 1,445.3, *p* < 0.01; *η* ^*2*^ = 0.73). Additionally, the results of multiple comparisons indicated significant differences in 30–39 years, in 40–49 years and in 50–59 years in comparison with the preceding age groups (Table 3). The 25th–75th percentile range for VFA was 37.9–125.6 cm^2^.

## Discussion

In this cross-sectional study, we assessed the age-related pattern of changes in body fat tissue (BFM and VAT) and FFM in adult Czech women. Although there are several studies that address similar topics [7–9], data describing the current body composition of adult women, including elderly women, are still lacking in the Czech Republic. Moreover, the Czech Republic is among the European countries with the highest prevalence of overweight and obesity [16], and therefore, these data are necessary not only to evaluate the current status of the problem but also to establish national guidelines and evidence-based prevention programs.

In the Czech Republic, the last national anthropometry survey was conducted in the late 1990s. The group of interest consisted of individuals younger than 55 years old. In the past 20 years, most research has been focused on children and adolescents [21, 22]. However, some studies with small samples were conducted with older age groups, but the results were based on anthropometric characteristics, such as BMI and skin-fold measurements [23, 24] or waist and hip circumference and waist-to-hip ratio [25]. For this reason, our study enrolled adult women, including elderly women. It aimed to define body composition, estimated from BSM-BIA, and to evaluate the changes that occur with aging. We also developed age-specific percentile curves, based on data collected from 1,970 Czech women aged 18–89 years. Age-related changes in selected body composition parameters were analyzed between each 10-year age group and the preceding age group, except for the youngest participants. Because current data are available only for subjects under 18 years old, we consider it important to emphasize that the first age group does not include a full decade; it begins at age 18 to provide continuity with current age-specific trends among Czech women.

### Age-related changes in BFM and %BFM

Body fat mass is the most variable component of the human body. In women, age-related changes in total and regional adiposity have been observed mainly after menopause [5]. In general, BFM peaks between the fifth and seventh decades of life, and it then remains constant or decreases slightly [3, 6–9]. However, this decline does not appear to be large in magnitude.

Several longitudinal studies have reported the amount that BFM increases throughout the lifespan, but they have reported different results. The Fels Longitudinal Study of 108 white women demonstrated that BFM increases with age by an average of 0.41 kg per year [2]. The results of an eight-year longitudinal study showed that BFM increases by 0.15 kg per year in young women (<45 years old) and by 0.24 kg per year in women over 45 years old [26]. Kyle et al. [27] found a non-significant increase of 0.20 kg (female: 0.04 kg, male: 0.32 kg) in BFM in both genders over 1 year of follow-up, but there was a statistically significant increase of 0.62 kg in BFM (female: 0.44 kg, male: 0.75 kg) over 3 years of follow-up. Dey et al. [28] reported age-related changes in body composition in a study of eighty-seven randomly selected 75-year-old subjects. BFM increased only in the men (1.16 kg increase over 5 years), whereas BFM was stable in the women. Among women older than 60 years old, Hughes et al. [1] observed that BFM increased by 7.5 % per decade. Our results confirmed these findings, but in a lower range (Table 2).

There have been numerous cross-sectional studies on the pattern of changes in body adiposity. According to the results of the US National Health and Nutrition Examination Survey (NHANES), BFM and %BFM, estimated by dual energy X-ray absorptiometry, peaked at 32.2 kg and 42.4 %, respectively, between the sixth and seventh decades of life, and they declined slightly thereafter [6]. The cross-sectional study of 5,522 healthy Western European subjects by Kyle et al. [7] showed that BFM and %BFM, estimated using single frequency bioelectrical impedance analysis (50 kHz), increased progressively in women between the ages of 15 and 98. The maximum BFM was observed between 65–74 years, and it declined thereafter. BFM increased by an average of 1.4 kg per decade in women between 15 and 74 years old. Another cross-sectional investigation demonstrated age-related changes in body composition in an Italian population between 20–80 years old. In women, the mean BFM was 21.5 kg and revealed a peak in BFM in the sixth decade of life. In addition, BFM remained constant in the oldest age group, and it significantly increased for the 30–39 and 60–69 age groups compared to the preceding age groups [9]. In non-Hispanic white women, BFM increased by 2.7 kg per decade from 20 to 59 years of age, and afterward, it decreased by 1.9 kg per decade [8]. Compared to the studies mentioned above, our cross-sectional data indicated that BFM peaked in the seventh decade and increased by 2.58 kg per decade. Moreover, the women in our study tended to have more BFM than the sample of Western European women [7] and less BFM than the non-Hispanic white women [8] and the Italian women [9].

### Age-related changes in visceral adipose tissue

It is well established that regional differences in body fat distribution are reliable predictors of health complications. The central distribution of BFM (particularly VAT accumulation) seems to be a strong independent predictor of all-cause mortality in both females and males [14]. There is also growing evidence that VAT is associated with age. In a longitudinal study, Lara-Castro et al. [29] demonstrated that significant increases in VFA were associated with age. In addition, each 1 kg increase in BFM over a 4-year follow-up period resulted in a VAT increase of 2.4 cm^2^. Sugihara et al. [30] concluded that VAT is positively correlated with age in all of the BMI strata in both genders. Another cross-sectional investigation [31], which included 130 participants and assessed VAT by computed tomography, clearly showed that VAT increased by 2.43 cm^2^ each year. In this study, 1 year of increasing age was associated with a mean VAT increase of 2.28 cm^2^. Therefore, our findings align with the above-mentioned age-related trends in VAT accumulation, and our findings also indicated a statistically significant change in VFA between each age group. The greatest difference (30.9 cm^2^) was observed between women aged 40–49 years and women aged 50–59 years. The change in VAT was most likely due to hormonal changes in menopause. This finding supports several studies [4, 5] that have indicated a significantly greater amount of VAT in postmenopausal women compared to premenopausal women. Generally, younger women have a lower amount of VAT, and hence, they have a lower risk of health complications associated with the development of abdominal obesity.

### Age-related changes in FFM

There is considerable variation in the way FFM changes over time. The significant decrease in FFM that accompanies aging primarily occurs as a result of the losses in skeletal muscle mass, which contribute to frailty and may lead to the development of sarcopenia. The prevalence of sarcopenia increases with age, and those over 80 years old are most often affected [10]. We observed that FFM was significantly lower among women over 70 years old compared to the women in the preceding age group. Moreover, even though FFM decreased, body weight increased slightly, due to the increase in BFM. It may also be assumed that the continuation of this trend (decreasing FFM and increasing BFM) could result in both sarcopenia and obesity. According to Zamboni et al. [11], sarcopenic obesity in the elderly may potentiate each other, maximizing their effects on disability, morbidity and mortality.

In our investigation, the mean FFM was 45.8 kg, and it decreased slightly with age at an average rate of 0.92 kg per decade (i.e., 0.09 per year). The peak level of FFM was observed in the fourth decade, at a value of 48.4 kg. The NHANES results indicated that FFM increased from childhood, peaked between 40 and 59 years at 45.1 kg and declined thereafter [6]. Similar age-related trends in body composition were also confirmed for Western European women by Kyle et al. [7] and for non-Hispanic white women by Chumlea et al. [8]. In contrast, Coin et al. [9] concluded that FFM peaked in the third decade at 42.7 kg in Italian women aged 20–80 years. A longitudinal study [26] showed that FFM increased by approximately 0.07 per year in younger women (<45 years), whereas it decreased by 0.09 kg per year in older women (≥45 years). According to Guo et al. [2], FFM decreased with age at an average rate of 0.11 kg per year in healthy women aged 18–98 years.

### Limitations of the study

Several limitations of this study should be considered. This study was cross-sectional, and body composition is dependent on age; therefore, it may not provide the same results as a longitudinal or semi-longitudinal study. However, other longitudinal [2, 26, 27] and cross-sectional studies [6–9] have observed similar results that support our findings.

Although the study sample includes nearly two thousand participants, the unequal distribution of participants among the age groups may limit our statistical power. Moreover, the study participants were volunteers, and some of them were recruited from universities (students and employees), which may have resulted in a selection bias. Thus, the results may not be representative of the general population, especially for the older age groups. Further research should be conducted using randomized studies with a varied population sample. The relationships between age-related changes and other factors, such as physical activity, nutrition, socioeconomic characteristics, demographics and environmental factors, should also be considered.

The accuracy and precision of BSM-BIA is known to depend on the hydration of FFM. The proportion is conventionally set at approximately 73.2 %. The hydration of FFM increases slightly in old age, and therefore, the results of BSM-BIA analysis may be affected, especially in older subjects [32]. Although Ogawa et al. [19] noted that BSM-BIA using InBody 720 shows to be useful as a more convenient substitute for computed tomography when measuring VFA, study by Lee et al. [33] showed that VFA measured by BSM-BIA (InBody 720) was only significantly correlated with VFA measured by computed tomography in premenopausal adult women with a BMI less than 30 kg/m^2^. In our study, BMI values were less than 30 kg/m^2^ in all age groups (range 21.8–28.2 kg/m^2^); therefore, the VFA values presented should be considered valid. Furthermore, there are no existing studies that have presented reference values and specific percentile curves for VFA in women aged 18–89 years.

In conclusion, there was a statistically significant increase in BFM, %BFM and VFA as age increased, and the values reached their peak in women over 70. Even when FFM decreased slightly with age, body weight increased because of the increase in BFM. These body composition data are important and can be used for comparison, intervention and evaluation purposes. Future studies should be longitudinal in design, and they should evaluate the relationships between body composition and other factors that may affect age-related changes.
